# Does low-dose spironolactone increase cardiovascular protection in patients with chronic kidney disease?

**DOI:** 10.1093/ckj/sfaf038

**Published:** 2025-02-11

**Authors:** Clara García-Carro, Pantelis Sarafidis

**Affiliations:** Nephrology Department, San Carlos Clinical University Hospital; Madrid Complutense University, Madrid, Spain; 1st Department of Nephrology, Aristotle University of Thessaloniki, Hippokration Hospital, Thessaloniki, Greece

Chronic kidney disease (CKD) is an expanding health problem globally. Both decreased kidney function, measured by estimated glomerular filtration rate, and kidney injury, measured by elevated albumin-to-creatinine ratio (ACR), are independent risk factors for cardiovascular events and mortality [1]. Despite this, the particularly high cardiovascular risk of patients with CKD, classic nephroprotective agents (i.e. angiotensin-converting enzyme inhibitors [ACEis] and angiotensin II receptor blocker [ARBs]), were not shown to reduce mortality in any major kidney outcome trial; in contrast, both sodium-glucose co-transporter-2 inhibitors (SGLT2is) and finerenone, a non-steroidal mineralocorticoid receptor antagonist (MRA), were shown to reduce cardiovascular events in albuminuric CKD patients [2].

Despite being effective in reducing albuminuria in CKD and cardiovascular outcomes in heart failure, older steroidal MRAs, such as eplerenone and spironolactone, have until recently never been tested in a trial with hard kidney or cardiovascular outcomes in patients with CKD. These knowledge gaps, in combination with the recorded risks of hyperkalemia and eGFR drop observed with these agents [3], were the reasons why they were never recommended for use in CKD patients. The BARACK-D trial was designed specifically to examine these important questions. It was a prospective, randomized, open, blinded end-point evaluation trial aiming to compare spironolactone 25 mg on top of usual care versus usual care alone in patients with 3b CKD with a mean age of 74.8 years, mean eGFR at baseline 43.5 mL/min/1.73 m^2^, and mean ACR 1.5 mg/mmol (A1). As expected, patients presented high rates of previous cardiovascular events. The primary outcome was a combination of cardiovascular events and death while secondary outcomes included changes in blood pressure, eGFR, ACR, hyperkalemia, and natriuretic peptide levels [4].

The trial included 1372 patients, of whom 677 were randomized to spironolactone and 695 to usual care alone. The median follow-up was three years. The number of patients who presented the primary cardiovascular outcome was not significantly different between the spironolactone and usual care arms at study end (16.7% versus 16%, respectively, *P* = 0.702). The death rate was also similar (6.2% versus 5.5%). On average, patients on spironolactone experienced a modest but significant reduction in eGFR when compared with the usual treatment group (adjustment treatment effect −1.14 mL/min/1.73 m^2^, *P* = .004), which initiated at month 6 and was stable until study end. ACR levels showed no difference between groups, and slightly worsened in both arms. At 1 year of follow-up, both systolic blood pressure and natriuretic peptide levels were significantly lower in the spironolactone group, but this difference was not present at study end [4].

Even more interesting is the high rate of study drug withdrawal in the spironolactone arm, as 67.2% of patients had treatment withdrawn at a mean time of 3.2 months after randomization. Of the withdrawals, 35.4% were because of eGFR decrease to a prespecified safety level, 8% due to hyperkalemia, and 19% due to other side effects. Hyperkalemia was more frequent with spironolactone (24.7% vs 13.4%, *P* < .001), and 4 patients presented with severe hyperkalemia in the intervention vs 0 in the usual treatment arm. Hypotension episodes were also more frequent with spironolactone [4].

An initial assessment of the study could make one think that the extremely high percentage of study drug withdrawal during the first months had greatly reduced the exposure time of the patients to this agent, and thus severely reduced the true statistical power, making it impossible for the study to offer any conclusions. However, in our opinion the authors have performed a technically solid study, incorporating safeguarding rules for study drug stop that are clinically relevant. Another important advantage is the inclusion of a population of old patients with stage 3b, normoalbuminuric CKD, a very common group in everyday clinical practice, especially in primary care, that has almost never been studied in kidney outcome trials. Overall, from a clinical point of view, the balanced conclusion is rather that spironolactone did not reduce cardiovascular events and death in this cohort, and was also poorly tolerated, being associated with more adverse events than usual treatment. In other words, this pragmatic study confirmed the reluctance of guideline texts and everyday doctors to advocate the use of spironolactone or eplerenone in CKD patients. Accordingly, spironolactone should rather not be used in patients with CKD without another specific indication (i.e. heart failure with reduced ejection fraction, primary aldosteronism, or resistant hypertension in patients with eGFR >45 mL/min/1.73 m^2^).

In view of these results, one must not forget that finerenone, a non-steroidal MRA, has proven efficacy in reducing both kidney and cardiovascular outcomes and safety in patients with type 2 diabetes, moderately or severely increased albuminuria and eGFR >25 mL/min/1.73 m^2^ in two major trials, FIGARO-DKD [5] and FIDELIO-DKD [6]. The reasons for these difference could be related to different pharmacological properties of steroidal and non-steroidal MRAs, as well as differences in the studied populations, as shown in Fig. 1, which compares baseline characteristics from the BARACK-D trial and FIDELITY pooled analysis [7]. An ongoing study, FIND-CKD, is currently testing the effects of finerenone in patients with albuminuric, non-diabetic CKD [8]. Thus, aldosterone antagonism remains a valuable pillar to improve outcomes in our patients and treatment recommendations to use finerenone in albuminuric diabetic CKD should be clearly followed [9].

**Figure 1:**
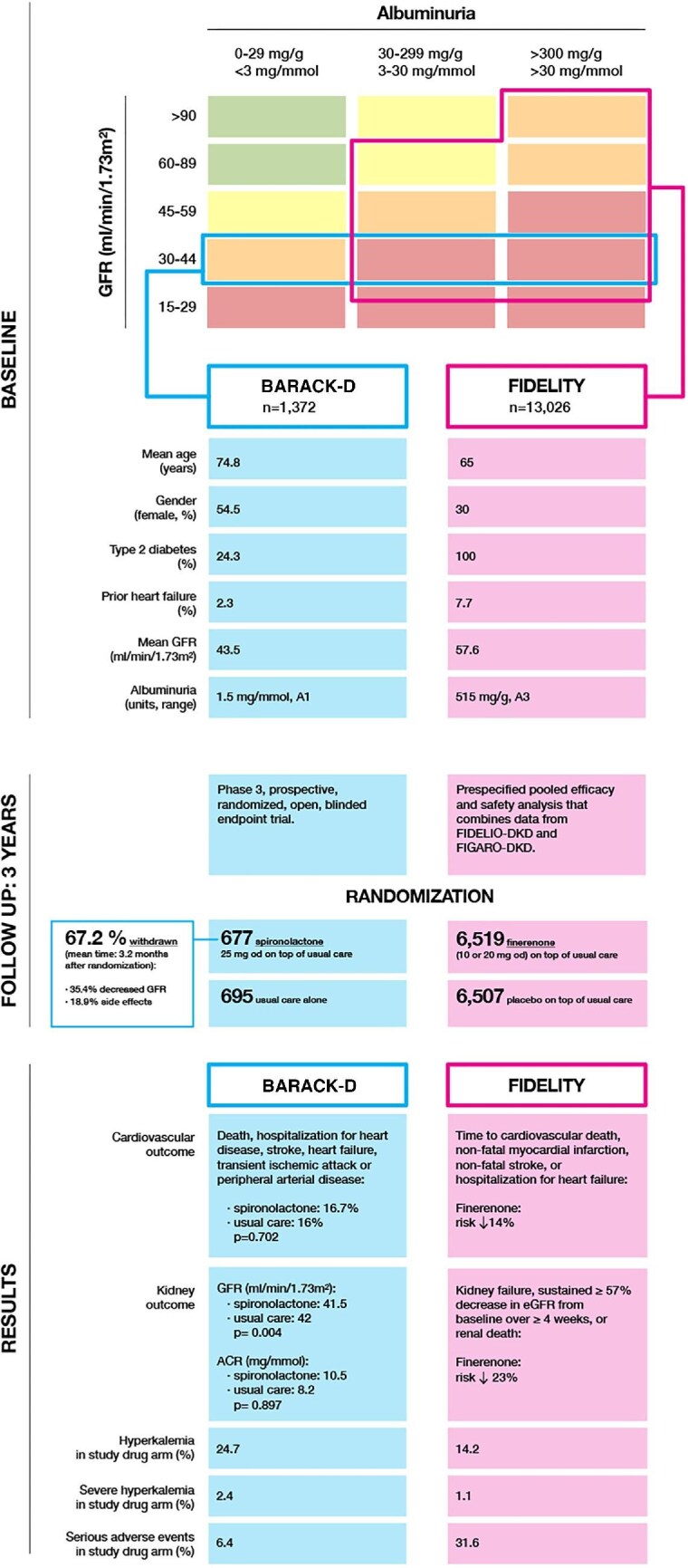
BARACK-D and FIDELITY studies: summary and comparison.

## References

[1] Fox CS, Matsushita K, Woodward M et al. Associations of kidney disease measures with mortality and end-stage renal disease in individuals with and without diabetes: a meta-analysis. Lancet 2012;380:1662–73. 10.1016/S0140-6736(12)61350-6 23013602 PMC3771350

[2] Sarafidis P, Papadopoulos CE, Kamperidis V et al. Cardiovascular protection with sodium-glucose cotransporter-2 inhibitors and mineralocorticoid receptor antagonists in chronic kidney disease: a milestone achieved. Hypertens 2021;77:1442–55. 10.1161/HYPERTENSIONAHA.121.17005 33775130

[3] Alexandrou ME, Papagianni A, Tsapas A et al. Effects of mineralocorticoid receptor antagonists in proteinuric kidney disease: a systematic review and meta-analysis of randomized controlled trials. J Hypertens 2019;37:2307–24. 10.1097/HJH.0000000000002187 31688290

[4] Hobbs FDR, McManus RJ, Taylor CJ et al. Low-dose spironolactone and cardiovascular outcomes in moderate stage chronic kidney disease: a randomized controlled trial. Nat Med 2024;30:3634–45. 10.1038/s41591-024-03263-5 39349629 PMC11753262

[5] Pitt B, Filippatos G, Agarwal R et al. Cardiovascular events with finerenone in kidney disease and type 2 diabetes. N Engl J Med 2021;385:2252–63. 10.1056/NEJMoa2110956 34449181

[6] Bakris GL, Agarwal R, Anker SD et al. Effect of finerenone on chronic kidney disease outcomes in type 2 diabetes. N Engl J Med 2020;383:2219–29. 10.1056/NEJMoa2025845 33264825

[7] Agarwal R, Filippatos G, Pitt B et al. Cardiovascular and kidney outcomes with finerenone in patients with type 2 diabetes and chronic kidney disease: the FIDELITY pooled analysis. Eur Heart J 2022;43:474–84. 10.1093/eurheartj/ehab777 35023547 PMC8830527

[8] Heerspink HJL, Agarwal R, Bakris GL et al. Design and baseline characteristics of the Finerenone, in addition to standard of care, on the progression of kidney disease in patients with Non-Diabetic Chronic Kidney Disease (FIND-CKD) randomized trial. Nephrol Dial Transplant 2024;40:308–19. 10.1093/ndt/gfae132 PMC11852274 38858818

[9] Sarafidis P, Schmieder R, Burnier M et al. A European Renal Association (ERA) synopsis for nephrology practice of the 2023 European Society of Hypertension (ESH) Guidelines for the Management of Arterial Hypertension. Nephrol Dial Transplant 2024;39:929–43. 38365947 10.1093/ndt/gfae041 PMC11139525

